# Differences In Antipsychotic Prescriptions In Relationship To Physician Demographics And In-patient Setting At An Inner-city hospital – A Prospective Cohort Study

**DOI:** 10.1192/j.eurpsy.2023.1172

**Published:** 2023-07-19

**Authors:** R. S. Ghatoura, J. Bhela, C. Trevino

**Affiliations:** 1 Psychiatry, Bronxcare Health System, The Bronx, United States; 2 Psychiatry, Windsor University School of Medicine, Cayon, Saint Kitts and Nevis; 3 Psychiatry, Essen Healthcare, The Bronx, United States

## Abstract

**Introduction:**

Antipsychotics are medications with an array of FDA approved indications in the field of psychiatry including Schizophrenia, Bipolar Mania, Bipolar Depression, Maintenance treatment for Bipolar Disorder, Schizoaffective Disorder, as well as an Adjunct treatment in Unipolar Depression and Tic disorders, among other indications for non-adult patients. Antipsychotics are widely used in psychiatric inpatient units throughout the United States, and globally. We observed trends of antipsychotic use in 3 different adult inpatient units with the same patient demographics located within one inner city hospital in the Bronx over a period of 4 months. We correlated the choice of antipsychotic to the prescribing physician’s period of training/date of graduation from psychiatry residency and reported the results.

**Objectives:**

Identify the choice of antipsychotics used by different psychiatrists.

Correlate the dates of each psychiatrists residency training to the antipsychotics they chose.

Identify whether the psychiatry residency training accuried in different decades has influenced psychiatrics to pick certain antipsychotics.

**Methods:**

We obtained the dates of psychiatric residency training for each of 3 psychiatrists (Physicians 1, 2 and 3) assigned to one of 3 different inpatient psychiatric units which share the same patient demographics in an inner-city hospital in the Bronx. We obtained a record of total psychiatric inpatient hospitalizations from February 10^th^ 2022 to June 10^th^ 2022 in all 3 psychiatric inpatient units. The principal diagnoses for the total hospitalizations (300 patients) were reviewed and patients with diagnoses that do not have FDA approval for antipsychotic use were excluded. Among the remaining patients (267) we compared antipsychotic prescription trends and grouped patients according to antipsychotic of choice. We then correlated the antipsychotic of choice groups to one of three units/prescribing physician and the years of psychiatry residency training. The trends for antipsychotic of choice were compared to training dates and presented in a table.

**Results:**

Physician 1 who trained from 1980 to 1984 prescribed: 87% HALDOL and 13 % Chlorpromazine. Physician 2 who trained from 1992 to 1996 prescribed: 91 % PALIPERIDONE and 9% Risperidone. Physician 3 who trained from 2003 to 2007 prescribed: 60 % ABILIFY and 40% Olanzapine.

**Conclusions:**

The physician with the earliest graduation date used mainly HALDOL, a first-generation antipsychotic, to treat disorders with FDA approval for antipsychotic use. The physician with the most recent graduation date, used mainly ARIPIPRAZOLE, a third-generation antipsychotic, to treat disorder with FDA approval for their use. The physician with a graduation date between them, used mainly (PALIPERIDONE), a second-generation antipsychotic to treat the disorders.

**Disclosure of Interest:**

None Declared

